# Perception of Iraqi Orthodontists and Patients toward Accelerated Orthodontics

**DOI:** 10.1155/2021/5512455

**Published:** 2021-04-29

**Authors:** Ali M. Al-Attar, Samher Al-Shaham, Mushriq Abid

**Affiliations:** Department of Orthodontics, College of Dentistry, University of Baghdad, Baghdad, Iraq

## Abstract

*Background*/*Purpose*. In the literature, no consensus about the duration of orthodontic treatment has been reached out. This study aimed to identify orthodontist's and patient's perception about the time of orthodontic treatment and their willingness to undergo and pay for various acceleration techniques and procedures. *Materials and Methods*. An electronic survey was conducted from August to October 2020. The questionnaire consisted of 20 multiple choice questions which was designed and emailed to members of the Iraqi Orthodontic Society and self-administered to patients in several orthodontic centers in Baghdad. The questionnaire included questions about the perception toward the duration of orthodontic treatment, approval of different procedures used to reduce treatment time, and how much fee increment they are able to pay for various techniques and appliances. Descriptive and chi-square test statistics were used, and the level of significance was set at *p* ≤ 0.05. *Results*. The response rate was 78.7%. The willingness for additional techniques and procedures was rated in the following order: customized appliances: 50.8% orthodontists and 38.4% patients, followed by intraoral vibrating devices: 49.2% orthodontists and 38.1% patients, piezocision: 10.2% orthodontists and 8.2% patients, and corticotomies: 8.1% orthodontists and 5.9% patients. Most orthodontists were willing to pay up to 40% of treatment income for the acceleration procedure, while the payment of patients was up to 20%. *Conclusion*. Both orthodontists and patients were interested in techniques that can decrease the treatment duration. Noninvasive accelerating procedures were more preferable by orthodontists and patients than invasive surgical procedures.

## 1. Introduction

A long term of orthodontic treatment is considered as a primary concern for most orthodontists and patients looking for treatment [1]. Long-term disadvantages of conventional orthodontic treatment such as predisposing the patient to caries, gingival recession, and resorption of roots had been a major concern to the patients. The essential objective of modern orthodontic treatment is to diminish the duration of orthodontic treatment by maximizing the biological response [2]. There could be a common agreement that the rate of tooth movement is controlled by the rate of bone resorption in the direction of tooth movement, which in turn is controlled by the rate of osteoclast differentiation and activation [3].

In an attempt to achieve rapid tooth movement, many researchers have used different approaches including chemical agents, such as prostaglandin E_2_ [4], calcitriol (active form of vitamin D_3_) [5–7], and hormones [8]. Moreover, physical agents such as electric currents [9], electromagnetic fields [10], vibrating devices [11], and low-level laser therapy [12, 13] or combination of two techniques have been used to accelerate tooth movement [14]. Moreover, besides these techniques, there have been significant improvements in the biomechanical behavior of fixed appliance brackets such as design, prescription, and material. This contributed to the evolution of several low-friction systems; however, treatment duration reduction is still debatable [15, 16]. Additionally, customized orthodontic brackets, archwire materials, and anchorage control have been reported to have clinical significance [17].

Several studies were performed on pain perception accompanied with acceleration techniques; most of them found that pain increased slightly in the first few days and then returned to normal with no significant differences between groups [18, 19]. Many factors determine the attitude of patients toward orthodontic treatment including age, gender, educational level, cost of treatment, and income level [20–23]. Patients were ready to pay up to 20% increase in fee for any procedure to reduce the treatment time [21].

No previous study in Iraq has investigated orthodontist's and patient's information and attitude toward orthodontic acceleration procedures. For this reason, this study was planned to determine orthodontists' and patients' perspectives toward orthodontic treatment time and procedures for accelerating the rate of tooth movement. Furthermore, the study was planned to evaluate the most accepted acceleration technique chosen by the orthodontists and patients and how much addition in fees they are willing to pay.

## 2. Materials and Methods

An electronic survey was designed and sent to orthodontists who are active members in the Iraqi Orthodontic Society (IOS) and self-administered to patients attending several orthodontic centers in Baghdad city by the researcher (A. A). The survey lasted twelve weeks from August 2020 till the end of October 2020. Orthodontists were asked to fill out an online questionnaire (Google Form) of twenty questions, while patients were asked to fill out a self-administered questionnaire with the same twenty questions. Participants were encouraged to contact the authors for inquiries related to answering the questionnaire. The procedure and protocol of the present study were approved by the College of Dentistry, University of Baghdad, in accordance with the Helsinki Declaration for human research studies.

The size of the sample was measured using the following formula:

(1) $$\begin{array}{c} N=\frac{\left(N/1\right)Z2xP\left(P-1\right)}{E2N}, \end{array}$$

where *N* is the size of the population, *Z* is the *z*-score for % confidence interval, *E* is the margin of error, and *P* is the population proportion (0.5). So, the estimated sample size for orthodontists was equal to 205 and 337 for patients at 95% confidence interval and 5% margin of error. To overcome dropout, additional 15% (31 orthodontists and 51 patients) was added to the sample; so, the final sample size was 236 orthodontists and 388 patients.

The questionnaire of this survey was adapted from two earlier studies [21, 24], and it was adjusted to include additional aspects. The primary survey was pilot-tested on 20 experienced orthodontists and 30 patients; consequently, the questions were reviewed and modified to the final form of the questionnaire.

Orthodontists' questionnaire was dispersed via e-mail by the IOS to all 360 active members with a link through Google Form. A data sheet clarifying briefly the clinical procedures that may accelerate the rate of tooth movement was added to the questionnaire to ensure full understanding of the questions (Supplementary material S1). The surveys were sent to the individuals by means of emails at least three times to maximize the response rate.

The survey for orthodontists was composed of several questions: demographic data including gender, academic degree, and duration of experience; their willingness to use different acceleration procedures plus the maximum cost they are willing to pay for these procedures; and how much increase in fee would be accepted for different levels of acceleration (Supplementary material S2).

Patients' questionnaire included the same demographic data questions in addition to their willingness to undergo selective techniques and procedures available to reduce the treatment time and how much increase in the fees they are willing to pay for reducing the duration of treatment (Supplementary material S3).

Descriptive and inferential statistics were performed for analyzing the data. Descriptive statistics were used to define all categorical data in the form of counts and percentages. Chi-square was performed to test the significant differences between orthodontists' and patients' willingness for various acceleration techniques and procedures plus fee increase for time reduction; the significance level was set at *p* ≤ 0.05.

## 3. Results

Three hundred and sixty questionnaires were e-mailed to orthodontists; two hundred and thirty-six were completed giving a response rate of 78.7%. More than half of the participants were male (59.3%), and most of them had M.Sc. degree (65.3%); 37.7% of the orthodontists had less than five years of experience.

Most of the orthodontists' response in regard to their satisfaction about the duration of active treatment ranged from neutral (32.2%) to somewhat dissatisfied (30.5%). Custom-made appliances were more familiar for orthodontists than other techniques, and the majority (42.4%) considered 20%–30% reduction in treatment time which was attractive (Table 1).

The characteristics of patients are summarized in Table 2. Female were more than male (59.8% and 40.2%, respectively), and the majority of them had less than 4 years of college (68.0%) since half of them were below 18 years old. The economical level for most patients (47.2%) was between 6000 and 9000$.

Most of the patients felt that orthodontic treatment takes too long time (58.8%), and they expected their treatment to finish between 12 and 18 months (49.2%), while more than half (57.5%) wished to finish before six months.

Table 3 summarizes orthodontists' and patients' willingness to use different acceleration techniques and the percentage of surplus amount of money they are willing to pay to decrease the treatment duration. For customized appliances, orthodontists were somewhat willing, and they were ready to give 40% of fee for a product, while patients were very willing to use these appliances, and most of them were ready to pay up to 20% increase fee.

Both orthodontists and patients were somewhat willing to use intraoral vibrating devices, and both were ready to pay 20% extra fee.

For surgical acceleration, orthodontists were neutral toward corticotomies and willing to pay up to 40% of fee, while patients were somewhat not willing, and most of them limited the fee increase up to 20%. For the less-aggressive surgical procedure (piezocision), orthodontists were somewhat willing (49.2%), while patients were somewhat not willing (33.7%). Similarly, the same was reported with intraoral injected drugs except that patients were neutral in willingness.

A summary of orthodontists' and patients' willingness for 25%–30% reduction in treatment time is demonstrated in Table 4. Both orthodontists and patients were mostly willing to use custom-made appliances and less willing toward intraoral vibrating devices, while corticotomies showed lesser willingness with no significant difference between them. Piezocision and intraoral injected local drugs showed a significant difference between orthodontists and patients in their willingness toward reduction in treatment time. Most orthodontists preferred piezocision, while patients preferred locally injected drugs.

Table 5 shows that there was a significant difference between orthodontists' and patients' willingness to pay extra fee for reduction in the treatment time.

## 4. Discussion

Cost of treatment is an important concept in healthcare that might have a detrimental effect on the treatment time and efficiency. Shorter orthodontic treatment duration with less extra fee is the primary goal for both patients and orthodontists. The present study was planned to determine orthodontist's and patient's perception for time of orthodontic treatment and their willingness to undergo and pay for various acceleration techniques and procedures.

The results of the present study reported that the majority of orthodontists were males having a M.Sc. degree with less than five years of experience since the orthodontic branch recently became available for most dentists in Iraq.

Most orthodontists were neutral in regard to the time of active orthodontic treatment as they believed that orthodontic tooth movement is a biological process and needs time to occur. The response agreed with the results of Uribe et al. who found that 93% of American orthodontists were neutral or satisfied with duration of treatment [21].

Custom-made appliances were familiar for about half of the orthodontists followed by piezocision and corticotomies (23.6% and 16.9%, respectively). Surprisingly, these surgical procedures were more familiar than less-invasive techniques such as intraoral teeth vibrators (8.5%) which could possibly be attributed to the high cost and lack of these appliances in the Iraqi markets.

Interestingly, a majority of orthodontists considered 20–30% reduction in treatment time to be attractive as acceleration techniques. This low percentage is reasonable because most acceleration techniques have not yet proven to be effective in accelerating tooth movement, and there is a controversy about their effectiveness.

Most of the patients were females with age below 18 years, i.e., precollege age. Similarly, a previous research study reported that the majority of orthodontic patients were of middle age but with no gender difference [22]. Orthodontic treatment is a lasting procedure and needs up to two years to be completed [25]. More than half of the patients in the present study (58.8%) strongly agreed that time of treatment is too long and expected that the course of treatment lasts for one to one and a half years which agreed with the previous study [26]. Moreover, most patients wished if orthodontic treatment lasted for less than six months, which was also reported by previous studies [21, 24].

Regarding the willingness of orthodontists and patients to use different acceleration procedures, the results revealed that custom-made appliances, intraoral teeth vibrators, and intraoral injected local drugs were preferred by most orthodontists and patients, while the majority of them were unwilling for surgical procedures (piezocision and corticotomies). This result can be justified by the willingness of both participants to choose less-invasive procedures which consequently have few side effects; the same findings were reported by several studies [18, 21, 24, 27, 28].

Regarding the cost, all patients were willing to pay not more than 20% extra fee, while most orthodontists were willing to pay up to 40% for acceleration techniques or appliances (Table 3). The same ability was reported by Linjawi and Abushal [29].

When evaluating the preference of orthodontists and patients for 25% to 30% reduction in treatment time, by the use of noninvasive techniques such as custom-made appliances and intraoral teeth vibrators (Table 4), most of them were willing to use these techniques with no significant difference, while piezocision and drug injection were less preferred by the patients. Piezocision was more preferred than corticotomies for both orthodontists and patients. This might show a desire for less-invasive flapless surgical procedures as reported by other authors [25].

The financial aspect demonstrated that the greater reduction in treatment time provoked patients for more payment as well as orthodontists, but still, the majority of patients limited the fee increase to 20%; however, there were significant differences in the desired fee increment between orthodontists who more ready than patients (Table 5). It is worth mentioning that the result of the present study represents the perception of Iraqi orthodontists and patients toward accelerated orthodontics which could be affected by the economic status of the country.

## 5. Conclusions

1. Most orthodontists and patients were not satisfied with the time of treatment, and they considered it as a lengthy procedure
2. Customized appliances were the most preferred technique for reducing the treatment time by orthodontists and patients followed by vibrating devices and then surgical procedures
3. Piezocision and locally injected drugs were less preferred by patients than orthodontists in appliances and techniques that reduce orthodontic treatment time 25%–30%
4. Orthodontists were ready to pay up to 40% of their fee for acceleration appliances and techniques, while patients limited fee increment to 20%

## Figures and Tables

**Table 1:** Characteristics of the orthodontists participating in the study.

Characteristic	Response	No. (%)
Gender	Male	140 (59.3%)
	Female	96 (40.7%)

Scientific degree	PhD	9 (3.8%)
	M.Sc.	154 (65.3%)
	Diploma/certificate	73 (30.9%)

Practice duration	<5 years	89 (37.7%)
	6–10 years	66 (28.0%)
	11–15 years	45 (19.1%)
	>15 years	36(15.3%)

Satisfaction of orthodontists with the amount of time patients are in active appliances	Very satisfied	24 (10.2%)
	Somewhat satisfied	64 (27.1%)
	Neutral	76 (32.2%)
	Somewhat dissatisfied	72 (30.5%)
	Very dissatisfied	0%

Familiarity of orthodontists with different techniques used for acceleration	Custom-made appliances	116 (49.2%)
	Intraoral teeth vibrators	20 (8.5%)
	Corticotomies	40 (16.9%)
	Piezocision	56 (23.7%)
	Locally injected intraoral drugs	4 (1.7%)

How much reduction in treatment time would you consider to use any acceleration technique?	0%–10%	12 (5.1%)
	10%–20%	36 (15.3%)
	20%–30%	100 (42.4%)
	30%–40%	56 (23.7%)
	Greater than 40%	32 (13.6%)

**Table 2:** Characteristics of the patients participating in the study.

Characteristic	Response	*n* (%)
1. Gender	Male	156 (40.2%)
	Female	232 (59.8%)

2. Education	Less than 4 years of college	264 (68.0%)
	Four years of college	106 (27.3%)
	Postgraduate degree	18 (4.6%)

3. Age	≤18 years old	195 (50.3%)
	>18–25 years old	171 (44.1%)
	>25–45 years old	22 (5.7%)

4. Annual income (ID)	<3000$	43 (11.1%)
	3000–6000$	102 (26.3%)
	>6000–9000$	183 (47.2%)
	>9000$	60 (15.4%)

5. Orthodontic treatment takes too long	Strongly agree	228 (58.8%)
	Somewhat agree	114 (29.4%)
	Neutral	34 (8.8%)
	Somewhat disagree	9 (2.3%)
	Strongly disagree	3 (0.8%)

6. How long do you expect your orthodontic treatment to take?	<12 mos.	103 (26.5%)
	12–18 mos.	191 (49.2%)
	18–24 mos.	94 (24.2%)

7. How long would you wish your orthodontic treatment to last?	<6 mos.	223 (57.5%)
	6–12 mos.	104 (26.8%)
	12–18 mos.	34 (8.8%)
	18–24 mos.	27 (6.9%)

**Table 3:** Willingness to use or undergo and pay for different procedures.

Appliance or technique	Group	Willingness to use/undergo/pay, *n* (%)					% of treatment fee increase, *n* (%)		
		Very willing	Somewhat willing	Neutral	Somewhat not willing	Not willing	0%–20%	20%–40%	Above 40%
Customized appliances	Orthodontists	40 (16.2%)	120 (50.8%)	56 (23.7%)	20 (8.5%)	0	100 (42.4%)	128 (54.2%)	8 (3.4%)
	Patients	149 (38.4%)	115 (29.6%)	89 (22.9%)	26 (6.7%)	9 (2.3%)	225(58%)	142 (36.6%)	21 (5.4%)

Intraoral vibrating devices	Orthodontists	12 (5.1%)	116 (49.2%)	44 (18.6%)	28 (11.9%)	36 (15.3%)	142 (60.2%)	94 (39.80%)	0
	Patients	95 (24.5%)	148 (38.1%)	82 (21.1%)	41 (10.6%)	22 (5.7%)	278 (71.6%)	98 (25.3%)	12 (3.1%)

Corticotomies	Orthodontists	19 (8.1%)	78 (33.1%)	89 (37.7%)	29 (12.3%)	21 (8.9%)	88 (37.3%)	136 (57.6%)	12 (5.1%)
	Patients	23 (5.9%)	45 (11.6%)	65 (16.8%)	201 (51.8%)	54 (13.9%)	264 (68.0%)	109 (28.1%)	15 (3.9%)

Piezocision	Orthodontists	24 (10.2%)	116 (49.2%)	64 (27.1%)	20 (8.5%)	12 (5.1%)	96 (40.7%)	124 (52.5%)	16 (6.8%)
	Patients	32 (8.2%)	46 (11.9%)	92 (23.7%)	129 (33.7%)	89 (22.9%)	243 (62.6%)	126 (32.5%)	19 (4.9%)

Locally injected intraoral drugs	Orthodontists	8 (3.4%)	96 (40.7%)	80 (33.9%)	8 (3.4%)	44 (18.6%)	104 (44.1%)	120 (50.8%)	12 (5.1%)
	Patients	45 (11.6%)	78 (20.1%)	109 (28.1%)	91 (23.5%)	65 (16.8%)	223 (57.5%)	142 (36.6%)	23 (5.9%)

**Table 4:** Preference for different procedures for a 25% to 30% reduction in treatment time.

Treatment	Group	Preference no. (%)					Chi-square	d.f.	*p* value
		1 (most willing)	2	3	4	5 (least willing)			
Custom-made appliances	Orthodontists	100 (42.4%)	56 (23.7%)	52 (22.0%)	20 (8.5%)	8 (3.4%)	3.435	4	0.487
	Patients	158 (40.7%)	128 (33.0%)	56 (14.4%)	28 (7.2%)	18 (4.6%)			

Intraoral teeth vibrators	Orthodontists	40 (16.9%)	108 (45.8%)	53 (22.5%)	19 (8.1%)	16 (6.8%)	7.354	4	0.11832
	Patients	113 (29.1%)	167 (43.0%)	45 (11.6%)	42 (10.8%)	21 (5.4%)			

Corticotomies	Orthodontists	48 (20.3%)	40 (16.9%)	64 (27.1%)	60 (25.4%)	24 (10.2%)	7.457	4	0.11362
	Patients	46 (11.9%)	62 (16.0%)	89 (22.9%)	102 (26.3%)	89 (22.9%)			

Piezocision	Orthodontists	40 (16.9%)	104 (44.1%)	48 (20.3%)	36 (15.3%)	8 (3.4%)	17.868	4	0.00131*∗*
	Patients	64 (16.5%)	78 (20.1%)	108 (27.8%)	82 (21.1%)	56 (14.4%)			

Locally injected intraoral drugs	Orthodontists	56 (23.7%)	44 (18.6%)	48 (20.3%)	64 (27.1%)	24 (10.2%)	11.751	4	0.019302*∗*
	Patients	43 (11.1%)	52 (13.4%)	87 (22.4%)	109 (28.1%)	97 (25.0%)			

*∗*Significant at *p* ≤ 0.05.

**Table 5:** Fee increase for reduction in time.

Reduction in time (%)	Group	Increase in fees by 10%	Increase in fees by 20%	Increase in fees by 30%	Increase in fees by 40%	Increase in fees by 50%	Chi-square	d.f.	*p* value
		No. (%)	No. (%)	No. (%)	No. (%)	No. (%)			
10	Orthodontists	20 (8.5%)	40 (16.9%)	20 (8.5%)	16 (6.8%)	16 (6.8%)	24.515	4	0.00006*∗*
	Patients	223 (57.5%)	86 (22.2%)	48 (12.4%)	22 (5.7%)	9 (2.3%)			

20	Orthodontists	72 (30.5%)	144 (61.0%)	72 (30.5%)	60 (25.4%)	40 (16.9%)	9.261	4	0.05489
	Patients	126 (32.5%)	142 (36.6%)	66 (17.0%)	33 (8.5%)	21 (5.4%)			

30	Orthodontists	128 (54.2%)	48 (20.3%)	128 (54.2%)	72 (30.5%)	60 (25.4%)	27.944	4	0.0000128*∗*
	Patients	88 (22.7%)	141 (36.3%)	87 (22.4%)	52 (13.4%)	20 (5.2%)			

40	Orthodontists	8 (3.4%)	0 (0.0%)	8 (3.4%)	80 (33.9%)	64 (27.1%)	76.174	4	*p* ≤ 0.001
	Patients	76 (19.6%)	119 (30.7%)	98 (25.3%)	62 (16.0%)	33 (8.5%)			

50	Orthodontists	8 (3.4%)	4 (1.7%)	8 (3.4%)	8 (3.4%)	56 (23.7%)	45.39	4	*p* ≤ 0.001
	Patients	74 (19.1%)	95 (24.5%)	102 (26.3%)	78 (20.1%)	39 (10.1%)			

*∗*Significant at *p* ≤ 0.05.

## Data Availability

The data used to support the findings of this study are made available from the corresponding author upon request.

## Supplementary Materials

Supplementary Materials S1: information sheet for orthodontists and patients. S2: questionnaire for orthodontists. S3: questionnaire for patients.
