# Supplementary material for: Perception of Iraqi Orthodontists and Patients toward Accelerated Orthodontics
Source: Int J Dent. 2021 Apr 29;2021:5512455. doi: 10.1155/2021/5512455 (PMC8102096; doi:10.1155/2021/5512455)
Supplement: Supplementary Materials — S1: information sheet for orthodontists and patients. S2: questionnaire for orthodontists. S3: questionnaire for patients. [file 5512455.f1.zip › 5512455.f1/S2.docx]

Questionnaire for Orthodontists

**All information of this survey will be used for academic purpose. Your responses will be anonymous and any personal information will be dealt with confidentiality. If you are willing to participate in this survey, please tick the box**

1-Gender

- Male
- Female

2-Scientific degree

- Ph.D.
- M.Sc.
- Diploma/Certificate

3. What kind of brackets do you use?

- Self-ligating
- Conventional
- Combination but with more self-ligating
- Combination but with more conventional

4. Please rate your overall satisfaction with the amount of time your patients are in active appliances.

- Very satisfied
- Somewhat satisfied
- Neutral
  - Somewhat dissatisfied
  - Very dissatisfied

5. Do you know about any of these techniques/technologies that reduce orthodontic treatment time?

- Custom-made appliances
- Piezocision
- Corticotomies
- Intraoral teeth vibrators
- Locally injected intraoral drug

6. How much reduction in treatment time would you consider attractive to use any of the above methods?

- 0%-10%
- 10%-20%
- 20%-30%
- 30%-40%
- 40%-50%
- 50%-60%
- Greater than 60%

7. How willing would you be to pay a company for special customized appliance that might reduce treatment time by 25% to 30%?

- Strongly willing
- Somewhat willing
- Neutral
- Somewhat not willing
- Not willing at all

8. How much of your treatment fee would you be willing to pay to a company for these special customized orthodontic appliances?

- 0%-20%
- 20%-40%
- above 40%

9. How willing would you be to pay a company for an intraoral vibrating device that might reduce treatment time by 25% to 30%?

- Strongly willing
- Somewhat willing
- Neutral
- Somewhat not willing
- Not willing at all

10. How much of your treatment fee would you be willing to pay to a company for this vibrating device?

- 0%-20%
- 20%-40%
- above 40%

11. How much would you be willing to pay a company from your treatment fee for custom-made appliances, intraoral vibrators, or other appliances, if they were able to reduce treatment time by 50%?

- 0%-20%
- 20%-40%
- above 40%

12. How willing are you to use corticotomies to significantly reduce treatment time?

- Strongly willing
- somewhat willing
- Neutral
- Somewhat not willing
- Not willing at all

13. If you would like to use corticotomies, how much increase in your treatment fee do you plan?

- 0%-20%
- 20%-40%
- above 40%

14. How willing are you to use piezocision to significantly reduce treatment time?

- Strongly willing
- Somewhat willing
- Neutral
- Somewhat not willing
- Not willing at all

15. If you are willing to use piezocision, how much increase in your treatment fee do you plan?

- 0%-20%
- 20%-40%
- above 40%

16. How willing are you to inject an intraoral local drug to significantly reduce treatment time?

- Strongly willing
- Somewhat willing
- Neutral
- Somewhat not willing
- Not willing at all

17. If you are willing to use local injectable intraoral drugs, how much increase in your treatment fee do you plan?

- 0%-20%
- 20%-40%
- above 40%

18. How strongly do you agree that reducing treatment time could become a problem in your treatment fee collections?

- Strongly agree
- Somewhat agree
- Neutral
- Somewhat disagree
- Strongly disagree

19. Please rank 1 through 5 based on your willingness to use these techniques in your practice (1 is most willing, 5 is least willing).

|  | 1 | 2 | 3 | 4 | 5 |
| --- | --- | --- | --- | --- | --- |
| Corticotomies |  |  |  |  |  |
| Piezocision |  |  |  |  |  |
| Intraoral teeth vibrator |  |  |  |  |  |
| Customized appliances |  |  |  |  |  |
| Intraoral injected drug |  |  |  |  |  |

20. Please check the appropriate fee increase for a particular treatment-time reduction

| Reduction in time | Increase in fees by 10% | Increase in fees by 20% | Increase in fees by 30% | Increase in fees by 40% | Increase in fees by 50% |
| --- | --- | --- | --- | --- | --- |
| 10% |  |  |  |  |  |
| 20% |  |  |  |  |  |
| 30% |  |  |  |  |  |
| 40% |  |  |  |  |  |
| 50% |  |  |  |  |  |
