# Supplementary material for: Perception of Iraqi Orthodontists and Patients toward Accelerated Orthodontics
Source: Int J Dent. 2021 Apr 29;2021:5512455. doi: 10.1155/2021/5512455 (PMC8102096; doi:10.1155/2021/5512455)
Supplement: Supplementary Materials — S1: information sheet for orthodontists and patients. S2: questionnaire for orthodontists. S3: questionnaire for patients. [file 5512455.f1.zip › 5512455.f1/S3.docx]

**Questionnaire for patients**

**All information of this survey will be used for academic purpose. Your responses will be anonymous and any personal information will be dealt with confidentiality. If you are willing to participate in this survey, please tick the box**

**1-Gender**

- Male
- Female

**2-Age**

- ≤18 years old
- >18–25 years old
- >25–45 years old
- >45 years old

**3-Education**

- Less than 4 years of college
- Four years of college
- Postgraduate degree

**4-Annual income (ID**)

- <3000$
- 3000-6000$
- 6000-9000$
- >9000$

**5-How strongly do you agree that orthodontic treatment takes too long?**

- Strongly agree
- Somewhat agree
- Neutral
- Somewhat disagree
- Strongly disagree

**6-How long do you expect your orthodontic treatment to take?**

- <12 mos.
- 12-18 mos.
- 18-24 mos.
- >24 mos.

**7-How long would you wish your orthodontic treatment to last?**

- <6 mos.
- 6-12 mos.
- 12-18 mos.
- 18-24 mos.
- >24 mos.

**8-Please rate how willing you would be to pay for customized appliance** that **reduces treatment time by 30%.**

- Very willing
- Somewhat willing
- Neutral
- Somewhat unwilling
- Very unwilling
- **9-If yes, what percentage of increase in your orthodontic treatment fee would you be willing to pay for these customized appliance?**
- 10%
- 20%
- 30%
- 40%
- 50%

**10-Please rate how willing you would be to use an FDA-approved teeth vibrator to reduce your treatment time.**

- Very willing
- Somewhat willing
- Neutral
- Somewhat unwilling
- Very unwilling
- **11-If yes, what percentage of increase in your orthodontic treatment fee would you be willing to pay to use this appliance?**
  - 10%
  - 20%
- 30%
- 40%
- 50%

**12-Please rate how willing you would be to undergo a one-time surgical procedure in addition to your braces to reduce treatment time (corticotomy).**

- Very willing
- Somewhat willing
- Neutral
- Somewhat unwilling
- Very unwilling
- **13-If you would be willing, what percentage of increase in your orthodontic treatment fee would you be willing to pay for this additional treatment?**
- 10%
- 20%
- 30%
- 40%
- 50%

**14-Please rate how willing you would be to undergo a one-time procedure to place small incisions (cuts) on the gums to reduce treatment time (piezocision) (see information sheet).**

- Very willing
- Somewhat willing
- Neutral
- Somewhat unwilling
- Very unwilling
- **15-If you would be willing, what percentage of increase in your orthodontic treatment fee would you be willing to pay for this additional treatment?**
- 10%
- 20%
- 30%
- 40%
- 50%

**16-Please rate how willing you would be to take an FDA-approved medicine injected around your teeth to reduce your treatment time (see information sheet).**

- Very willing
- Somewhat willing
- Neutral
- Somewhat unwilling
- Very unwilling
- **17-If you would be willing, what percentage of increase in your orthodontic treatment fee would you be willing to pay for this additional treatment?**
- 10%
- 20%
- 30%
- 40%
- 50%

**18-Would you be able to pay higher monthly payments to reduce your treatment time?**

- Very able
- Somewhat able
- Neutral
- Somewhat unable
- Very unable

**19-Please rank 1 through 5 these additional orthodontic techniques or procedures in the order you prefer or feel would be convenient (1 signifies most willing through 5, which signifies unwilling) to undergo if advised considering a 25% to 30% reduction in treatment time.**

- Customized appliance _____
- Teeth vibrator----------------
- Corticotomies (bone cuts) ---------------
- Piezocision (cuts on the gums) ----------------
- Drug injected around the teeth ----------------

**20. Please check the appropriate fee increase for a particular reduction in treatment time**

| Reduction in time | Increase fees by 10% | Increase fees by 20% | Increase fees by 30% | Increase fees by 40% | Increase fees by 50% |
| --- | --- | --- | --- | --- | --- |
| 10% |  |  |  |  |  |
| 20% |  |  |  |  |  |
| 30% |  |  |  |  |  |
| 40% |  |  |  |  |  |
| 50% |  |  |  |  |  |

**Additional comments**

**Thank you for completing our survey.**
