# Supplementary material for: Perception of Iraqi Orthodontists and Patients toward Accelerated Orthodontics
Source: Int J Dent. 2021 Apr 29;2021:5512455. doi: 10.1155/2021/5512455 (PMC8102096; doi:10.1155/2021/5512455)
Supplement: Supplementary Materials — S1: information sheet for orthodontists and patients. S2: questionnaire for orthodontists. S3: questionnaire for patients. [file 5512455.f1.zip › 5512455.f1/S1.doc]

**Information Sheet for Orthodontists and patients**

Tittle: **Orthodontists and Patients Perception to Accelerated Orthodontics**

- This questionnaire-based research study intends to investigate how patients and orthodontists perceive additional treatment procedures that might reduce the time that patients are in braces.
- This survey includes questions about how much the participants would be willing to pay or charge for these additional treatment procedures that can reduce orthodontic treatment time.

Before making a decision to participate in this research study, please read this information sheet carefully.

***The questionnaire does not contain any identifiable or personal information.*** Please do not put any identifiable information anywhere in the questionnaire.

**Information about the additional procedures discussed in the questionnaire to reduce orthodontic treatment time**

| **Procedure** | **Description** | **Photographs** |
| --- | --- | --- |
| **Corticotomies**  **Intraoral vibrating device**  **Custom-made appliances**  **Piezocision**  **Intraoral injected drug** | Cuts are made on the bone surrounding the teeth after raising a gum flap (picture) to increase the rate of tooth movement.  A device to be used in the mouth 10 to 20 minutes daily to vibrate the teeth with the orthodontic appliances.  All appliances used are custom made for the patient according to his/her treatment plan.  Instead of reflecting a flap, small microcuts are made through the gums to increase the rate of tooth movement.  .  Drugs injected around the teeth as shown in the picture. |          |
